# A Scoping Review of Heteronormativity in Healthcare and Its Implications on the Health and Well-Being of LGBTIQ+ Persons in Africa

**DOI:** 10.3390/ijerph22050717

**Published:** 2025-05-01

**Authors:** Lebogang Manthibe Ramalepe, Tshepo B. Maake

**Affiliations:** 1 Compres Research Entity, North West University, Vanderbijlpark 1900, South Africa; 2 Department of Sociology, School of Social Sciences, University of South Africa, Pretoria 0003, South Africa; emaaketb@unisa.ac.za

**Keywords:** heteronormativity, healthcare, LGBTIQ+ persons, SDG 3, SDG 10, Africa

## Abstract

This scoping review explores the patterns of heteronormativity in healthcare sectors across Africa and its impact on the health and well-being of LGBTIQ+ individuals. By analyzing publications from major academic databases, this study identifies key themes and research gaps in the discourse on LGBTIQ+ health and heteronormativity in Africa. The findings underscore the urgent need to address these heteronormative barriers in alignment with the United Nations Sustainable Development Goals (SDGs), particularly SDG 3 (good health and well-being) and SDG 10 (reduced inequalities). Addressing heteronormativity in healthcare is essential for promoting equitable, inclusive healthcare systems and improving health outcomes for LGBTIQ+ populations across the continent.

## 1. Introduction

A substantial body of literature on sexualities in Africa has established that LGBTIQ+ identities are often exposed to intolerance and limited acceptance due to the perpetuation of heteronormative ideologies [1,2,3,4]. The term heteronormativity was coined by Warner [5], who argued against the universalization of a heterosexual worldview that excluded possibilities of sexual diversity. Warner [5], in conjunction with Katz [6], questions the naturalization of heterosexuality by arguing that it is not a natural existence but a historically invented mode of sexual organization that was intended to be universal, unchanging, and essential. Heterosexuality, therefore, represents a specific sexual–political institution that establishes distinct expectations for its members and marginalizes those who fail to conform to dominant heterosexual norms [6]. Thus, the invention and universalization of the institution of heterosexuality informed the construction of compulsory heterosexual expectations within various social spaces, compelling individuals to be sexually attracted to members of the opposite sex and desire the heterosexual family organization [7]. In addition, the institution of compulsory heterosexuality prioritizes the reproductive nature of sexual relations, consequently excluding sexual behaviors that are non-reproductive, inter-alia same-sex sexual relations. Butler [8] teaches us that heterosexual norms are structured within a matrix that delineates a tripartite relationship between sex, sexual identity, and the gendered norms of masculinity and femininity, which are conventionally associated with specific bodies. Heteronormativity is, therefore, a sociocultural norm that recognizes heterosexuality as the only way to express a sexual identity, with no other possibilities [9]. Heteronormative ideologies emphasize the procreational character of sexual relationships, normalizing heterosexuality, while rendering other expressions of sexuality unnatural. Within a heteronormative framework, male and female sexual bodies are assumed to align with masculine and feminine gendered categories and the binary gendered desire for the opposite sex [8].

While the concept of heteronormativity was originally coined and first applied in the Global North, scholars from the Global South, particularly in Africa, have highlighted its relevance and significance, often demonstrating how it hinders the recognition of sexual minority identities and contributes to their exclusion and persecution in diverse contexts [10]. Dominant sexuality discourses in African countries are rooted in heteronormative worldviews that are guided by religious doctrines and African cultural traditions that are intolerant of sexual diversity [11]. The implementation of these anti-gay laws and punishments are informed by heteronormative religious doctrines, specifically from the Christian and Islamic religions, which most African subscribe to. They are also driven by the idea that African cultural traditions do not recognize sexual relations between men or women.

These heteronormative ideologies have contributed to legal frameworks and social structures that marginalize LGBTIQ+ individuals across the African continent. A key argument among many African societies is that diverse sexual identities are Western imports that never existed in Africa [12,13,14]; however, some scholars challenge this claim, by demonstrating that sexual minority identities have always existed in Africa, even before the colonization of most African countries and were incorporated into African cultural traditions and social structures in various ways [1,2,3,4]. In their writings, these scholars highlight historical accounts, indigenous practices, and oral traditions that acknowledge same-sex desires and non-binary gender identities and challenge the view that diverse sexual identities are foreign to African societies. They instead argue that the colonization of Africa, and the imposition of Western morality and legal systems, led to the suppression and criminalization of alternative expressions of gender and sexuality in Africa [4,9,15]. Additionally, religious ideologies, particularly Christian and Muslim, have been used to argue against same-sex sexual desires and relationships, which has informed unjust legal frameworks and policies that ban alternative expressions of sexuality in many African countries [14,16]. Colonial legal and religious systems played a significant role in the oppression and criminalization of sexual minority identities in many African countries [1,3,17,18], indicating the degree of intolerance and lack of acceptance of sexual diversity across the continent. These unjust legal frameworks inform the stigmatization, discrimination, and exclusion of LGBTIQ+ individuals in various social contexts, including homes, schools, workplaces, religious institutions, universities, and the health sector [19]. Thus, heteronormative ideologies are embedded in these institutions, limiting the expression of sexually diverse identities and limited access for LGBTIQ+ communities. Although access to quality healthcare services is considered a basic human right globally, the international literature has established that for LGBTIQ+ people, access is often hindered by heteronormative ideologies and cultures that do not recognize sexual diversity. This is particularly evident in African countries where sexual minority individuals are exposed to homophobic violence and intimidation [10,16]. While some African countries, such as South Africa, legally prohibit the discrimination of people based on their sexual identities, research has established that heteronormativity is still evident and forms part of the workplace cultures in healthcare institutions, affecting how LGBTIQ+ people are treated when they seek medical attention [20,21]. Inclusive legal frameworks, such as those of South Africa and Mozambique, two of the few countries in Africa that fully recognize sexual diversity and protect the rights of sexual and gender minorities, do not guarantee the elimination of heteronormative expectations that dictate how people should express their sexual identities and perform their gender identities. As such, many LGBTIQ+ people in Africa live in fear and may avoid seeking medical assistance, especially for illnesses that require them to disclose their sexual identities [21,22]. The hindrance and limited access to healthcare services significantly affects the wellbeing of sexual minority individuals who are vulnerable to mental health issues, Sexually Transmitted Infections (STIs) and Human Immunodeficiency Virus (HIV). The increased burden of HIV and STIs amongst the LGBTIQ+ community is also informed by the heteronormative barriers to access and use of HIV and STI prevention services in different African countries [19,23]. Heteronormative ideologies inform medical training and health policies, often neglecting the unique health needs of LGBTIQ+ individuals. The absence of inclusive healthcare structures exacerbates disparities in mental health, HIV prevention, and access to sexual and reproductive health services for sexual minorities [24,25]. Irrespective of the growing recognition of these issues, no comprehensive review has examined the extent of heteronormativity in African healthcare systems. Previous reviews such as those conducted by Müller & Hughes [26] and Newman-Valentine & Duma [27] have addressed specific aspects of LGBTIQ+ health, particularly in South Africa. However, none of these reviews explored the broader patterns of heteronormativity in healthcare institutions across Africa. This scoping review seeks to address this gap by mapping the existing literature on the social and structural barriers that LGBTIQ+ people encounter in healthcare settings across various African countries.

The social exclusion and lack of recognition of LGBTIQ+ people in health facilities undermine international efforts towards achieving the United Nations Sustainable Development Goals (UNSDGs), particularly SDG 3 on good health and well-being and SDG 10 on reduced inequalities. The primary aim of this scoping review is to identify and summarize the literature on the impact of heteronormativity on the healthcare experiences of sexual minority individuals in Africa. Exploring the various ways in which heteronormative ideologies inform policies, medical training, and healthcare providers’ interactions with patients helps to bring forth the barriers LGBTIQ+ individuals encounter in healthcare. Consequently, this scoping review seeks to answer the question: What is the scope and nature of the existing literature on heteronormativity in African healthcare settings? Ultimately, this scoping review focuses on identifying key themes, research gaps, and policy implications in the literature, with the goal of informing future interventions and reforms that are aimed at promoting inclusive healthcare.

The heterogeneity of laws regulating LGBTIQ+ rights in various African countries presents challenges in drawing broad conclusions. In some countries, sexual minority identities are criminalized and punishable by law, while others offer varying degrees of legal recognition and protection (1,17,18]. In Nigeria, Uganda, and Ghana, homosexuality is criminalized and punishable by imprisonment and, in extreme cases, the death penalty [17,28]. Countries such as Tanzania and Sudan have also employed strict anti-LGBTIQ+ laws that contribute to the continuing social stigma that leads to the avoidance of healthcare services due to fear of discrimination [29]. In contrast, South Africa has progressive legal frameworks that protect sexual minority rights; however, studies have demonstrated that heteronormative biases persist in healthcare settings, affecting access to quality healthcare services [20,21]. Same-sex relationships were decriminalized in Mozambique in 2015 through its revised penal code, yet some reports indicate that LGBTIQ+ people still experience discrimination when seeking healthcare services [30]. Botswana has made significant strides in recognizing sexual minority rights by decriminalizing same-sex relationships in 2019, but systematic challenges remain in healthcare institutions [28]. This scoping review acknowledges these differences and considers how legal contexts inform the healthcare experiences of sexual minority individuals. Where necessary, the literature will be contextualized to reflect variations in healthcare access based on the legal and social contexts of these African countries. In examining the existing literature on heteronormativity in African healthcare institutions, this scoping review highlights the urgent need to address discriminatory practices that hinder sexual minority individuals from accessing equitable healthcare. Addressing heteronormativity in healthcare is crucial to fostering inclusive and non-discriminatory healthcare environments that uphold the rights and dignity of all individuals, irrespective of their gender and sexual identities.

## 2. Materials and Methods

### 2.1. Review Typology, Research Question, Aims, and Justification

A scoping review methodology was chosen to address the research question: What is the scope and nature of the existing literature on heteronormativity in African healthcare settings? Scoping reviews are effective for broadening the understanding of topics that have yet to be fully explored, as they systematically chart the existing body of evidence and identify gaps in the knowledge [31,32]. In the context of heteronormativity and healthcare in Africa, this approach allows for a comprehensive overview of how societal norms surrounding sexuality influence healthcare systems, practices, and outcomes, with particular attention to how these factors affect LGBTIQ+ populations. Given that heteronormativity is a key determinant of health inequities, especially in contexts where LGBTIQ+ individuals may face legal and social stigma, mapping out the existing literature will offer valuable insights into the scope and depth of research in this area.

This scoping review is guided by the six-stage framework developed by the Joanna Briggs Institute [33], which provides a systematic process for identifying, selecting, and synthesizing relevant studies. The stages involve defining the research question, identifying relevant studies, selecting studies for inclusion, charting the data, collating and summarizing the findings, and finally, reporting the results. Through this approach, this study will not only assess the available literature but will also help to identify critical gaps in the knowledge, providing a foundation for future research aimed at addressing the health disparities faced by LGBTIQ+ communities in Africa. By mapping the literature on heteronormativity in healthcare, this study will contribute to broader discussions on healthcare equity and the steps needed to help African countries achieve SDG 3 and SDG 10, promoting access to respectful and inclusive healthcare for all populations, regardless of gender and sexual identity.

### 2.2. Eligibility Criteria and Search Strategy

To gather the relevant academic documents for this study, the researchers employed a systematic approach utilizing a variety of reputable databases, including Scopus, Google Scholar, PsycInfo, and Web of Science. These databases were chosen for their comprehensive access to a wide range of scholarly sources, such as peer-reviewed journal articles, theses, book chapters, and books, all of which met the study’s inclusion criteria. The scoping review specifically focused on studies conducted in Africa, this is mainly due to Africa’s distinct socio-cultural and political contexts, which has been shaped by colonial histories, religious conservatism, and legal barriers, significantly influence LGBTQI+ healthcare experiences [34]. Limiting the review to this region ensures contextually relevant insights into these unique challenges. To maintain academic rigor, only peer-reviewed articles are included, as they undergo expert evaluation, enhancing the credibility and reliability of findings [31]. Additionally, restricting the review to English-language articles ensures accessibility and consistency in analysis, particularly due to the researchers lacking proficiency in other languages in Africa such as French, Portuguese, and Spanish [35]. While this may exclude some perspectives, English remains the universal language in academic publishing, ensuring broad coverage of the relevant literature.

The search strategy was designed to capture a broad spectrum of the relevant literature by utilizing specific keywords aligned with the research objectives. Key search terms included “heteronormativity”, “healthcare”, “LGBTQI”, and “Africa”. Boolean operators (“AND” and “OR”) were strategically applied to refine the search and ensure that only the most pertinent documents were selected. The review focused on the literature published within the past 19 years (2005–2024) to capture the latest insights into the role of heteronormativity in healthcare settings and inform future research directions and potential interventions. The primary search keywords included heteronormativity, healthcare or health, LGBTQI or lesbian or gay or bisexual or transgender or queer or intersex, and Africa. To enhance the search strategy and ensure broader coverage, secondary search keywords incorporated heteronormativity, healthcare or health, LGBTQI or lesbian or gay or bisexual or transgender or queer or intersex, along with Africa and its regional subdivisions: Southern Africa, West Africa, East Africa, North Africa, and Central Africa. This approach aimed to capture relevant studies across different geographical contexts and terminologies. Table 1 and Table 2 below outlines the search terms that we used and the eligibility criteria that were followed in the selection of studies that were relevant to the scoping review.

### 2.3. Study Selection and Data Extraction

Once the search process was completed, all identified studies were imported into Covidence™ for further analysis. Covidence™ automatically removed any duplicate entries, ensuring that only unique studies were considered. The screening process was carried out in two stages: title and abstract screening, followed by full-text screening. During each stage, studies were excluded if it was determined that they did not meet the pre-established eligibility criteria. To ensure consistency and minimize bias, two authors independently screened the articles at both stages. In cases of disagreement, the issue was resolved through discussion or by consulting a third author. The final study selection process was documented using the Preferred Reporting Items for Systematic Reviews and Meta-Analyses (PRISMA 2020) flow diagram [36]. This systematic approach to study selection helped to maintain transparency and rigor throughout the review process.

### 2.4. Data Analysis

This study utilized Reflexive Thematic Analysis (RTA), as outlined by Braun and Clarke [37], to investigate key themes and patterns within the literature on heteronormativity and healthcare in Africa. This approach was well-suited to the research aims, as it supports the systematic identification of recurring ideas while aligning with the interpretivist framework that informs this study [38]. RTA enables an in-depth exploration of how heteronormative structures influence healthcare policies, access, and experiences, particularly for sexual and gender minorities. By emphasizing a flexible and iterative engagement with data, this method allows themes to emerge organically rather than being confined to predefined categories, facilitating a deeper understanding of the relationship between heteronormativity and healthcare in African contexts.

## 3. Results

**Figure 1:**
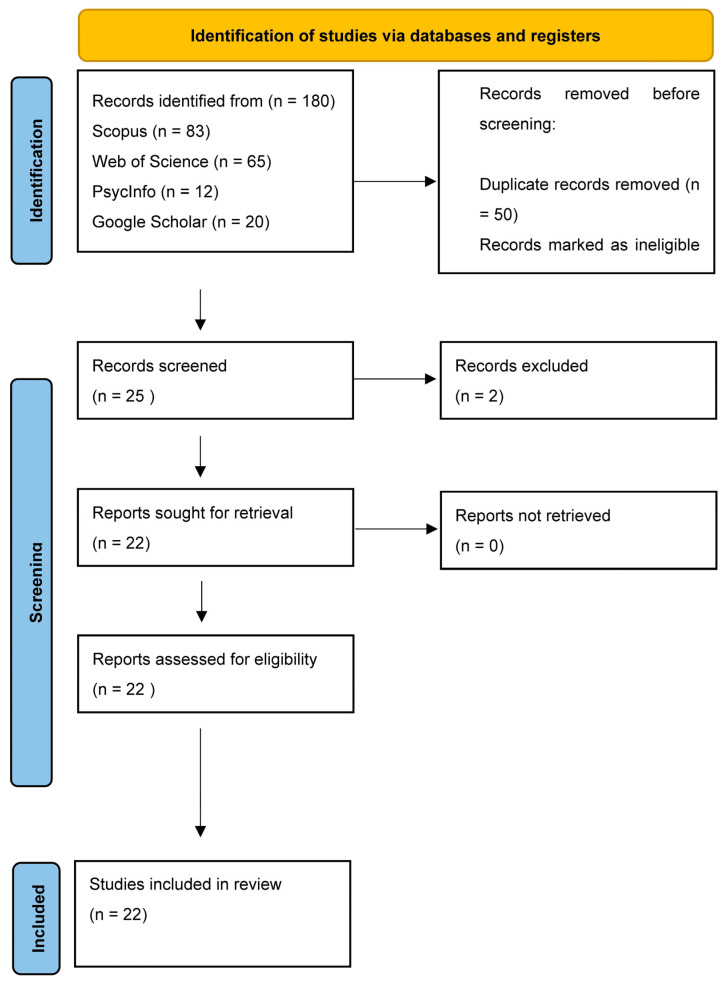
PRISMA flow diagram.

As shown in Figure 1, only 180 articles were identified and imported into Covidence. A total of 22 papers met our eligibility criteria.

### Characteristics of Included Studies

Of the 22 included publications, 21 were primary research papers, while 2 were review papers. Of these, 22 were journal articles, and 1 was a PhD thesis. Most of the studies (*n* = 16) were conducted in South Africa. One paper included studies conducted in Tanzania, Zimbabwe, Ethiopia, and Nigeria, respectively, while another focused on five countries in Southern Africa, with notable representation from three French-speaking countries: Côte d’Ivoire, Cameroon, and Senegal. Overall, the studies included in this review represented regions in Southern, Western, and Eastern Africa. The participants mainly consisted of LGBTQI individuals in Africa.

## 4. Findings

The following themes were identified:

### 4.1. Theme 1: Heteronormativity and Structural Barriers in Healthcare

Healthcare spaces in many African contexts are structured around policies that fail to recognize or address the specific health needs of LGBTIQ+ individuals. As scholars such as Ratele [39] and Currier [24] have noted, the absence of inclusive health policies reflects broader socio-political structures that marginalize queer identities, often portraying them as deviant or un-African. These policies contribute to the systemic exclusion of LGBTIQ+ individuals from mainstream healthcare services, reinforcing heteronormativity as the standard for health and well-being. Furthermore, the historical and cultural framing of queerness as pathological—rooted in colonial-era laws and religious dogma—continues to shape contemporary healthcare practices, leading to the invisibilization of sexual and gender minorities within national health systems. This exclusion forces LGBTIQ+ individuals to either forego essential healthcare or seek services in environments where they may not feel safe or affirmed.

The pervasive stigma and discrimination experienced by LGBTIQ+ individuals in healthcare settings have far-reaching consequences on their health-seeking behaviors and overall well-being. Studies by Müller [34] and Fay et al. [40] illustrate how men who have sex with men (MSM) and transgender persons, in particular, face prejudicial treatment from healthcare providers, often resulting in the denial of services, substandard care, or outright hostility. Such discrimination discourages LGBTIQ+ individuals from accessing healthcare facilities, leading to delays in seeking treatment and increased health vulnerabilities. This aligns with research by Logie et al. [41], which demonstrates how stigma in healthcare settings exacerbates health disparities among LGBTIQ+ populations, particularly in relation to HIV prevention, mental health services, and sexual and reproductive healthcare. The intersection of institutional bias and individual prejudice creates a healthcare environment that is not only exclusionary but also actively harmful to LGBTIQ+ persons who require competent and affirming medical attention.

The marginalization of LGBTIQ+ individuals within public healthcare systems is further compounded by their invisibility in health education and training programs. Research by Epprecht [1] has highlighted how medical and nursing curricula in many African countries lack substantive content on LGBTIQ+ health, leaving healthcare providers ill equipped to provide competent care. This lack of education reinforces discriminatory attitudes among health professionals, as seen in campus healthcare settings where LGBTIQ+ students report experiencing significant prejudice from university medical staff [42]. As a result, LGBTIQ+ individuals are often forced to develop subversive strategies to access care, such as withholding information about their sexual or gender identities to avoid mistreatment. However, as Mogotsi, I., et al. [43] argue, these coping mechanisms are unsustainable and place the burden of navigating discriminatory systems on individuals rather than prompting necessary structural reforms.

### 4.2. Theme 2: Barriers to Healthcare Access for LGBTIQ+ Individuals and Its Impact

LGBTIQ+ individuals face significant structural and social barriers to healthcare access, often driven by stigma, discrimination, and legal restrictions. Fear of disclosure due to stigma prevents many from seeking timely medical care, as accessing services can expose them to harassment or legal repercussions in contexts where homosexuality is criminalized [41]. Healthcare access is frequently contingent on conforming to heteronormative norms, forcing individuals to suppress their identities to receive appropriate care [34]. Transgender men and “tomboys” experience additional obstacles, including outright denial of services, while women who have sex with women (WSW) often encounter healthcare professionals who dismiss their sexual and reproductive health needs due to misconceptions about their risk factors [24,44]. Economic constraints, fear of being outed, shame, and direct refusal of care further limit healthcare access, compounding the effects of systemic exclusion [20]. Additionally, rejection by family and community intensifies social isolation, leading to poor mental health outcomes, including depression and engagement in risky sexual behavior as a coping mechanism [43]. The lack of robust social support systems further exacerbates these vulnerabilities, reinforcing cycles of health inequities [45]. Addressing these disparities requires comprehensive policy reforms, LGBTIQ+ inclusive healthcare training, and legal protections to dismantle the barriers that sustain health inequities for LGBTIQ+ populations.

### 4.3. Theme 3: The Role of Healthcare Providers in LGBTIQ+ Health Outcomes

Despite the growing recognition of LGBTIQ+ health needs, gender-affirming care remains largely unavailable or dependent on the discretion of individual healthcare providers, as institutional support and policy frameworks for such services are often lacking [44]. The absence of structured training in LGBTIQ+ health results in a widespread lack of competence among healthcare professionals, reinforcing discriminatory attitudes and exclusionary practices [34]. Many healthcare providers operate with deeply ingrained biases, which manifest as gatekeeping behaviors, refusal of care, or the imposition of heteronormative treatment approaches that fail to address the unique health concerns of sexual and gender minorities [43]. This institutionalized discrimination underscores the urgent need for comprehensive professional training and sensitization programs that equip healthcare workers with the knowledge and skills necessary to provide inclusive, nonjudgmental, and affirming care to LGBTIQ+ individuals [25]. Without such systemic interventions, LGBTIQ+ individuals will continue to face barriers to essential healthcare services, exacerbating health disparities and reinforcing cycles of marginalization and inequality.

### 4.4. Theme 4: The Role of Law, Policy, and Institutional Structures

Legal frameworks that criminalize homosexuality significantly shape healthcare access and treatment for LGBTIQ+ individuals, reinforcing stigma and deterring them from seeking necessary medical care [40]. Weak policy enforcement mechanisms fail to protect sexual and gender minorities from discrimination within healthcare settings, leaving them vulnerable to mistreatment by healthcare providers who may hold prejudiced views [34]. The African human rights system has largely been ineffective in addressing violations of LGBTIQ+ healthcare rights, as regional institutions often defer to national sovereignty on matters related to sexual and gender identities [46]. Policy reforms that explicitly recognize and integrate LGBTIQ+ health needs into national health agendas are essential for fostering gender-sensitive and inclusive healthcare services [47]. Structural changes within healthcare institutions—such as provider sensitization programs, the inclusion of LGBTIQ+ health in medical curricula, and the implementation of affirmative policies—are crucial to creating safer and more equitable healthcare environments [25]. Strengthening legal protections for LGBTIQ+ persons, alongside intentional policy shifts, is fundamental to ensuring their right to health and combating systemic discrimination in healthcare across the African continent [48].

### 4.5. Theme 5: The Need for Inclusive Medical Curriculum and Training

Medical and nursing curricula across Africa largely exclude comprehensive LGBTIQ+ health content, leaving healthcare professionals unprepared to address the specific health needs of sexual and gender minorities [34]. The absence of structured training on LGBTIQ+ health perpetuates gaps in knowledge, reinforcing discriminatory attitudes and limiting access to appropriate care [49]. Additionally, existing curricula fail to create spaces where students can critically engage with and challenge their biases, resulting in healthcare providers who may consciously or unconsciously uphold heteronormative healthcare practices [9]. Given the disproportionate burden of HIV, mental health challenges, and sexual health disparities within LGBTIQ+ communities, integrating these topics into medical education is essential for fostering inclusive and competent healthcare systems [50]. Training programs that explicitly address the intersection of sexuality, gender identity, and health can enhance provider competence and ensure that LGBTIQ+ individuals receive affirming and equitable care [24].

### 4.6. Theme 6: The Intersection of Colonialism, Religion, and LGBTIQ+ Health

Colonial-era laws and religious doctrines continue to play a significant role in the oppression of LGBTIQ+ individuals in Africa, often embedding discriminatory practices into societal structures [1]. These laws, combined with entrenched social norms rooted in colonial and religious values, perpetuate stigma and discrimination, particularly in healthcare settings, where LGBTIQ+ individuals often face barriers to access and quality care [51]. Addressing this structural discrimination requires dismantling the inherited oppressive legal frameworks and fostering a cultural shift toward inclusivity and equity in both public and private spheres [52]. Such reforms are essential for ensuring that LGBTIQ+ populations are protected and have equitable access to health and social services.

In Table 3 below, we provide a description of the thematic categories that were derived from data analysis. This is followed by Table 4, which provides brief descriptions of the articles that were selected and included in the scoping review.

## 5. Discussion

The findings from this scoping review highlight the pervasive heteronormative barriers LGBTIQ+ individuals encounter in accessing equitable healthcare in many African contexts. As noted in the themes, heteronormativity and structural barriers within healthcare systems significantly hinder the well-being of sexual and gender minorities. The lack of inclusive health policies, as identified by Ratele [39] and Currier [24], perpetuates systemic exclusion by reinforcing heteronormative standards that marginalize LGBTIQ+ individuals. This exclusion is further compounded by the legacy of colonial-era laws and religious doctrines, which continue to frame LGBTIQ+ identities as deviant or pathological. This historical context not only shapes public attitudes but also informs healthcare practices, leading to the invisibility of LGBTIQ+ individuals within national health systems [41]. Such exclusion forces LGBTIQ+ people to navigate unsafe healthcare spaces or forgo essential services, which exacerbates health disparities and increases vulnerability to conditions such as HIV, mental health issues, and sexual health complications.

The findings demonstrate that the stigma and discrimination that LGBTIQ+ individuals experience in healthcare settings further intensify these barriers to healthcare. Studies by Müller [34] and Fay et al. [40] demonstrate how healthcare providers often treat LGBTIQ+ individuals with hostility, resulting in the denial of care or substandard treatment. These discriminatory practices discourage LGBTIQ+ individuals from seeking medical attention, thereby delaying necessary interventions and increasing health risks. As Mogotsi, I. et al. [43] point out, individuals may adopt coping strategies, such as withholding information about their sexual orientation or gender identity, to avoid mistreatment. However, these strategies are unsustainable and place an unfair burden on individuals, rather than prompting necessary reforms to address the underlying biases within healthcare systems. Logie et al. [41] emphasize that stigma not only affects health-seeking behaviors but also exacerbates existing health disparities, particularly in areas like HIV prevention, mental health services, and sexual health care, where LGBTIQ+ individuals are already disproportionately affected.

The role of healthcare providers in shaping LGBTIQ+ health outcomes is critical, as evidenced by the limited availability of gender-affirming care and the lack of training on LGBTIQ+ health. Reisner et al. [44] and Müller [34] highlight that the absence of structured training programs in many African countries results in healthcare professionals being ill equipped to provide competent, inclusive care. This lack of training reinforces discriminatory attitudes and exclusionary practices that harm LGBTIQ+ individuals. As Poteat et al. [25] argue, without comprehensive professional training and sensitization programs, healthcare providers are likely to perpetuate institutionalized discrimination through gatekeeping behaviors and the imposition of heteronormative treatment approaches. The need for LGBTIQ+-inclusive medical curricula is further emphasized by Singh et al. [72], who stress that integrating LGBTIQ+ health issues into medical and nursing training is crucial to developing a healthcare workforce that is both competent and compassionate in providing care to sexual and gender minorities.

Legal frameworks and policies also play a significant role in shaping healthcare access for LGBTIQ+ individuals. The criminalization of homosexuality in many African countries, as discussed by Fay et al. [40] and Ekine and Abbas [46], not only stigmatizes LGBTIQ+ identities but also deters individuals from seeking necessary healthcare for fear of legal repercussions. Furthermore, limited policy enforcement, particularly in countries that legally recognize the rights of LGBTIQ+ individuals, often leaves LGBTIQ+ individuals vulnerable to discrimination within healthcare settings, where healthcare providers may act with impunity [34]. The African human rights system, according to Farmer, M. [52], has often failed to address these violations effectively, as regional institutions defer to national sovereignty on matters related to sexual orientation and gender identity. To disrupt these barriers, comprehensive policy reforms are necessary, including legal protections for LGBTIQ+ persons and the implementation of affirmative policies that promote inclusive healthcare. As Baral et al. [47] suggest, these reforms are essential for fostering gender-sensitive and inclusive healthcare services that address the specific needs of LGBTIQ+ populations and ensure their right to health and social services.

Ultimately, we argue that the available literature on heteronormativity in healthcare, although limited, highlights the pervasiveness of heteronormative ideologies and their limitations to healthcare for sexual and gender minorities. Much of the literature is situated in South Africa, which is a point of concern considering that most African countries do not acknowledge and protect the rights of LGBTIQ+ communities. While resistance from African governments to legally recognize LGBTIQ+ rights remains prevalent, we contend that fundamental human rights, particularly the right to access healthcare, must take precedence. Healthcare institutions in Africa should prioritize addressing the health needs of sexual and gender minorities, ensuring that care is provided without reinforcing heteronormative ideologies and expectations. While many African countries claim to be democratic states, heteronormative intolerance and the lack of acceptance both on a legal and social basis in many continues to undermine LGBTIQ+ individual’s right to access quality healthcare. Furthermore, it undermines international efforts, made through the SDGs to reduce inequalities and improve access to healthcare, affecting Africa’s progress in achieving these goals. These arguments underscore the need for systemic change within healthcare systems, including policy reforms, education, and training, to address the heteronormative barriers faced by LGBTIQ+ individuals in accessing equitable and inclusive healthcare across Africa.

## 6. Limitations

The scoping review was limited by the availability of academic sources within the selected databases, which may have affected the comprehensiveness of the included literature. As noted by Tricco et al. [73], database selection plays a crucial role in determining the breadth of literature included in a review, and focusing on specific databases may inadvertently exclude relevant studies. Moreover, studies published in languages not covered in this review could have been overlooked, reducing the study’s global scope [74]. Expanding language inclusion and diversifying database coverage in future research would help to mitigate this limitation and provide a more inclusive view of the topic.

## 7. Conclusions

This scoping review identified significant themes of heteronormativity in African health institutions. The themes unpacked in this article not only highlight the heteronormative barriers to healthcare in various African contexts but the urgency in dealing with these barriers that hinder LGBTIQ+ communities’ access to quality healthcare. It is concerning that even in a country such as South Africa, where sexual and gender minorities are protected by the constitution and other legal frameworks, LGBTIQ+ individual’s access to quality healthcare is hindered by heteronormative ideologies that are perpetuated and enforced on a social level by health practitioners. The primary aim of the Sustainable Development Goals (SDGs) is to transform our world into a better place, where everyone can enjoy good health, justice, and prosperity [75]. African nations must not selectively heed this call, but promote a complete transformation of their health systems, to ensure that people are not left behind or excluded based on how they identify their gender and sexual identities.

### Ethical Considerations

This scoping review adhered to ethical considerations, which are outlined in this section. Unlike primary research involving human participants, scoping reviews generally do not require ethical approval; however, researchers must ensure the accurate and unbiased selection, analysis, and reporting of the literature to maintain research credibility (Peters et al., 2020 [33]). This review exclusively included peer-reviewed journal articles, excluding the grey and unpublished literature, to uphold data reliability and consent principles (Tricco et al., 2018 [73]). Additionally, ethical principles such as proper citation, avoiding plagiarism, and preventing misrepresentation of findings were strictly followed throughout the research process (Arksey & O’Malley, 2005 [31]). Transparency in methodology was maintained, and this scoping review adhered to established frameworks, including those proposed by the Joanna Briggs Institute (JBI) and PRISMA-ScR guidelines, thereby strengthening its ethical rigor (Levac, Colquhoun, & O’Brien, 2010 [76]).

## Figures and Tables

**Table 1:** Search terms used.

List of the Search Items
Heteronormativity, healthcare or health, LGBTQI or lesbian or gay or bisexual or transgender or queer or intersex, Africa.
Heteronormativity, healthcare or health, LGBTQI or lesbian or gay or bisexual or transgender or queer or intersex, Africa, or Southern Africa, or West Africa, or East Africa, or North Africa or central Africa.

**Table 2:** Eligibility criteria.

Category	Inclusion Criteria	Exclusion Criteria
Context		
Topic	Studies explicitly focusing on heteronormativity in healthcare among LGBTQI+ individuals in Africa	Any study that did not focus on heteronormativity in healthcare among LGBTQI+ individuals
Source type	Peer-reviewed journal articles	Training manuals, social media, policy documents, books
Study design	All study designs	
Publication year	2005–2024	Post-2024 November
Language	English	Other languages with no English abstract

**Table 3:** Thematic categories derived from data analysis.

Thematic Categories	Secondary Themes	Tertiary Themes
***Theme 1: Heteronormativity and Structural Barriers in Healthcare***	*1.1 Heteronormativity in Healthcare Policies* *1.2 Stigma and Discrimination in Healthcare Settings* *1.3 Health Disparities and Barriers to Care* *1.4 Deficiencies in Medical Education and Training*	
***Theme 2: Barriers to Healthcare Access for LGBTIQ+ Individuals and its impact***	*2.1 Stigma, Discrimination, and Legal Barriers* *2.2 Heteronormativity in Healthcare Systems* *2.3 Unique Healthcare Barriers for Specific LGBTIQ+*	
***Theme 3: The Role of Healthcare Providers in LGBTIQ+ Health Outcomes***	*3.1 Lack of Institutional Support and Policy Frameworks* *3.2 Heteronormative Treatment Approaches* *3.3 Bias and Discriminatory Attitudes*	
***Theme 4: The Role of Law, Policy, and Institutional Structures***	*4.1 Weak Policy Enforcement and Discrimination* *Policy Reform for Inclusive Healthcare*	
***Theme 5: The Need for Inclusive Medical Curriculum and Training***	*5.1 Lack of LGBTIQ+ Health Education in Medical Training*	*5.1.1 Improvement of Cultural Competency in Healthcare* *5.1.2 Integration of Gender-Affirming Care in Medical Curricula* *5.1.3 Professional Development and Continuing Education*
***Theme 6: The Intersection of Colonialism, Religion, and LGBTIQ+ Health***	*6.1 Colonial Legacy and Criminalization of Homosexuality*	*6.1.1 Religious Doctrines and Health Stigma* *6.1.2 The Role of Religious Institutions in Healthcare*

**Table 4:** Overview of articles included in the scoping review.

Author, Year	Type of Publication	Geographical Location	Sample	Data Collected	Main Findings
Meer & Muller [53]	Journal article	South Africa	29 queer service-users and 14 representatives of an organization	Qualitative (in-depth interviews and focus groups)	Findings reveal that healthcare spaces are produced by the spatial ordering of health policy inattentive to queer health needs; the enduring symbolic representations of queerness as pathological or “un-African”; and various identity assertions and practices of individuals, including queer service-users and healthcare providers. As a result, healthcare spaces are overwhelmingly heteronormative, although queer service-users’ subversive practices suggest alternative spatial configurations. However, such resistance relies on individual empowered action and risks disciplinary responses. Wider efforts are needed to transform the material and ideological space of healthcare facilities through law and policy reform and continuing professional training for healthcare providers.
Mange et al. [54]	Journal article	South Africa	15 participants	Qualitative (in-depth interviews)	A key finding of the study was that the OBGM, who are people living with HIV, were stigmatized and faced discrimination from the healthcare professionals at the hospital. Rejection by their families and communities and the death of their life partners led to isolation and depression. Social workers should be involved in counselling OBGM, training healthcare professionals, and facilitating workshops with families and communities in the townships.
Morison & Lynch [55]	Journal article	South Africa	34 participants	Qualitative interviews	Findings show how sexual, and gender minorities are discursively invisibilized in health settings and discuss these findings in relation to the social justice and solidarity aims of health system reform.
Scherf et al. [56]	Journal article	South Africa	Literature review	Qualitative	The African system’s historical lack of effective response to LGBT rights violations in the African continent, alongside weak enforcement instruments, suggests that this may not be exactly the right forum to address the health rights violations of transgender people in South Africa.
Mulemfo et al. [57]	Journal article	South Africa	6 participants	Qualitative (interpretive phenomenological analysis)	The findings indicate that LGBTIQ+ people are marginalized, discriminated against, and stigmatized in the public PHC system, exposing them to unequal access to healthcare services. The heterocentric system prevents them from accessing specific HIV management services and appropriate preventive commodities. The study concludes that gender diversity, inclusion and sensitivity in healthcare provision, and specific LGBTIQ+ training for healthcare providers are crucial components of ensuring LGBTIQ+ people’s access to quality HIV management services.
Mkhize et al. [58]	Journal article	South Africa	12 participants (LGBT-identifying)	Qualitative	There is a need for a well-planned curriculum that includes LGBTIQA+ issues to equip healthcare professionals with the knowledge to provide high-quality care to all patients, regardless of their sex, gender, or sexuality.
Kamazima [59]	Journal article	Tanzania	12 participants	Cross-sectional descriptive and retrospective formative qualitative	Social and legal strictures against homosexuality, coupled with widespread heteronormativity, put women who have sex with women at risk of overt or covert stigma and discrimination in the healthcare system. The illegal status of homosexuality in this country shapes differentiated health-seeking behaviors and pathways among sexually minority women. Healthcare providers are reported to be discriminating against and stigmatizing transgender men and tomboys, forcing them to avoid visiting public health facilities.
Hunt et al. [60]	Journal article	Zimbabwe	6 participants	Qualitative study using in-depth interviews and focus groups, with thematic analysis	Participants described barriers to accessing even basic healthcare due to discrimination perpetrated by healthcare professionals. Equal access to care was dependent on conforming to “sexual norms”. Healthcare professionals’ personal attitudes affected care delivery, and key populations were perceived to have brought illnesses on themselves through sexual behavior.
Mavhandu-Mudzusi [20]	Journal article	South Africa	20 LGBT university students	Qualitative (interpretive phenomenological analysis)	The findings of the study focus on citizenship rights and the discrimination that LGBTI students experience in accessing healthcare services. The main forms of discrimination reported are the heterocentric nature of services and treatment at the campus health clinic and the heteronormative prejudice held by university healthcare personnel.
Spencer et al. [61]	Journal article	South Africa	12 healthcare providers	Qualitative	Findings suggest that, whilst a small minority of healthcare providers offer gender-affirming care, this is almost exclusively on their own initiative and is usually unsupported by wider structures and institutions. The ad hoc, discretionary nature of services means that access to care is dependent on whether a transgender person is fortunate enough to access a sympathetic and knowledgeable healthcare provider.
Muller [62]	Journal article	South Africa	Two case studies	Qualitative	Findings highlight the complex and intersecting discrimination and marginalization that sexual and gender minority individuals face in healthcare in this particular context. The issues raised in the case studies are not unique to South Africa, however; the human rights concerns illustrated therein, particularly around the right to health, have wide resonance in other geographical and social contexts.
Nhamo-Murire & Maclead [63]	Journal article	South Africa	Literature review	Qualitative	The results show a nexus of experiences of exclusion and oppressive social norms. Our analytical framework highlighted absences in nursing practice. No research indicates that LGB people experience nurses as advocates or in participatory healthcare processes.
Adekola [64]	Journal article	South Africa	Literature review	Qualitative	The results indicated that gender roles, cultural norms, heteronormativity, gender-based violence, and associated stigmatization are gender-specific barriers to adolescents’ health literacy in South Africa.
Muranda et al. [65]	Journal article	Africa	Literature review	Qualitative (feminist virtual ethnography)	The findings of this study highlight a tension that, on the one hand, exists between the heteronormativity of healthcare providers and broader society, and the ways in which this silences lesbians and other women who have sex with women in their healthcare interactions and, on the other, the totalizing view of WSW sexualities within this community, which silences conversations about HIV because such conversations may expose or accuse a person of “not being a real lesbian”. The women within the African lesbian, gay, bisexual, transgender, and intersex (LGBTI) community who participated in our study had scant access to credible HIV/AIDS and safe-sex information, resulting in various and dangerous (mis)conceptions proliferating. The vulnerability of lesbians and other WSWs to HIV infection is a complicated public health issue that is perplexing to some and ignored by many, not only on the African continent but globally.
Muller & Hughes [26]	Journal articles	Southern Africa	Literature review	Systematic review	Identifying large gaps in the literature, the review highlighted substantial sexual-orientation-related health disparities among women in Southern Africa. The findings have important implications for public health policy and research, highlighting the lack of population-level evidence on the one hand, and the impact of criminalizing laws around homosexuality on the other hand.
Newman-Valentine & Duma [27]	Journal articles	South Africa	10 transsexual women	Qualitative (interpretative phenomenological analysis)	This article highlights issues affecting the health and healthcare delivery for transgender women to assist healthcare practitioners to reflect on their automatic heteronormative healthcare practices. It also has implications for the promotion of inclusivity in the development of curriculum that shapes inclusive healthcare providers. The article will hopefully serve as a vehicle to mobilize researchers to investigate issues affecting the health and healthcare delivery for transsexual women within the African context.
Seretlo et al. [66]	Journal articles	South Africa	55 one-on-one interviews	Explorative–descriptive qualitative study	Six main themes emerged, demonstrating that HCPs and queer people faced similar, contrasting, and differing challenges when rendering and receiving SRHS. These themes include HCPs’ belief that queer people are afraid, while queer people perceive HCPs as having negative attitudes and acting as gatekeepers. HCPs expressed surprise and confusion regarding gender identity, healthcare disparities, and familial issues, which highlighted their feelings of incompetence in providing queer-related healthcare and their engagement with queer people as a barrier.
Kleinhans [67]	Journal articles	South Africa	5	Semi-structured, in-depth key informant interviews	The findings of this study show that LGBTI students are underserved in the campus healthcare system, and this is the result of a heteronormative campus environment.
Tadele & Made [68]	Journal articles	Ethiopia	118	Concurrent mixed-method design	The results show that heteronormativity intersects with LGB people’s social position (sexual identity, social network, and class) to influence healthcare needs, health-seeking behavior, or access to health services. Sexual health and mental health problems are the main concerns of LGB, who reported living under acute anxiety and fear of being exposed or bringing shame and humiliation to themselves or their families. One of the main emerging themes from the research is the link between mental health and risky sexual practices. The risk perception of HIV was high among LGB, with two-thirds reporting high risk. Only 37.5% (33/88) stated being always motivated to seek care when sick and the rest cited the following barriers that stifled their health seeking behavior and utilization of healthcare services: stigma and discrimination (83%), shame and embarrassment (83%), fear of being discovered (78%), lack of LGB-friendly services (45%), affordability (18%), distance (17%), and healthcare professional refusal (10%).
Müller [69]	Journal articles	South Africa	127 academic respondents	Survey	A total of 127 academics across 31 divisions and research units in the Faculty of Health Sciences responded to the survey, of which 93 completed the questionnaire. Ten taught some content related to LGBT health in the MBChB curriculum. No LGBT health-related content was taught in the allied health sciences curricula. The MBChB curriculum provided no opportunity for students to challenge their own attitudes towards LGBT patients, and key LGBT health topics such as safer sex, mental health, substance abuse, and adolescent health were not addressed.
Wiginton et al. [70]	Journal articles	Côte d’Ivoire, Cameroon, Lesotho, eSwatini, and Senegal	4405 respondents	Quantitative	These findings underscore the need for not only the eradication of all forms of stigma and discrimination in healthcare contexts but the presence of intentional affirmation of minority sexualities and marginalized persons. Supportive environments that enable safe disclosure are critical for providing appropriate sexual health services to MSM and maintaining their engagement in sexual health service utilization. For many MSM in countries across SSA, the disclosure of same-sex practices lies at a critical intersection of tradeoffs, potentially leading to improved social support and sexual healthcare services (e.g., HIV pre-exposure prophylaxis access; improved counselling tailored to same-sex practices/partnerships) but also to stigmatization and victimization. Preventing sexuality-based stigma in healthcare settings requires a fundamental change to heteronormative societal structures that perpetuate harmful norms and stereotypes and maintain policies that stigmatize sexual minority men. Ultimately, advancing the HIV response in SSA and around the world necessitates multilevel interventions to mitigate community, institutional, and interpersonal-level stigma; foster enabling environments for disclosure of same-sex practices; and facilitate access to broader social and extrafamilial supports for MSM who have experienced stigma.
Sekoni [71]	PhD Thesis	Nigeria		Mixed methods	The concept of the “hidden” speaks about the unseen or invisible aspects of LGBT experiences and explores what it takes for an LGBT person to seek healthcare beyond the normal psychological and emotional effort. It also looks at the damage inflicted on the individual following exposure to oppression from a healthcare provider. This storytelling is in the context of postcolonial Africa, where homosexuality is often considered un-African, the laws and religion inherited from that era form the basis for promulgating more stringent laws against LGBT people, and discrimination is a method for holding onto what is culturally African. Colonialism and laws, therefore, play an important role as social determinants of health. This study was able to bring to the fore the hidden fact that there are levels to the degree of disadvantage experienced by subgroups within the LGBT community. The extent of inequality is determined by the intersection of factors beyond the control of affected individuals.

## Data Availability

All data generated or analysed during this scoping review are derived from publicly available published sources, which are cited in the reference list.
